# A multi-strain probiotic blend reshaped obesity-related gut dysbiosis and improved lipid metabolism in obese children

**DOI:** 10.3389/fnut.2022.922993

**Published:** 2022-08-04

**Authors:** An-Chyi Chen, Tzu-Jung Fang, Hsieh-Hsun Ho, Jui-Fen Chen, Yi-Wei Kuo, Yen-Yu Huang, Shin-Yu Tsai, Shu-Fen Wu, Hung-Chih Lin, Yao-Tsung Yeh

**Affiliations:** ^1^Division of Pediatric Gastroenterology, China Medical University Children’s Hospital, Taichung City, Taiwan; ^2^School of Medicine, China Medical University, Taichung City, Taiwan; ^3^College of Medicine, Graduate Institute of Clinical Medicine, Kaohsiung Medical University, Kaohsiung City, Taiwan; ^4^Division of Geriatrics and Gerontology, Department of Internal Medicine, Kaohsiung Medical University Hospital, Kaohsiung City, Taiwan; ^5^Department of Research and Design, Glac Biotech Co., Ltd., Tainan City, Taiwan; ^6^Division of Neonatology, China Medical University Children’s Hospital, Taichung City, Taiwan; ^7^School of Chinese Medicine, China Medical University, Taichung City, Taiwan; ^8^Asia University Hospital, Asia University, Taichung City, Taiwan; ^9^Aging and Disease Prevention Research Center, Fooyin University, Kaohsiung City, Taiwan; ^10^Department of Medical Laboratory Science and Biotechnology, Fooyin University, Kaohsiung City, Taiwan

**Keywords:** childhood obesity, probiotics, gut microbiota, high-density lipoprotein (HDL), adiponectin

## Abstract

**Background and aims:**

Obese children are more prone to becoming obese adults, and excess adiposity consequently increases the risk of many complications, such as metabolic syndromes, non-alcoholic fatty liver disease, cardiovascular disease, etc. This study aimed to evaluate the effects of multi-strain probiotics on the gut microbiota and weight control in obese children.

**Methods:**

A double-blind, randomized, placebo-controlled trial was carried out on overweight and obese children. Subjects received 12 weeks of treatment with supplementary probiotics that contained three strains: *Lactobacillus salivarius* AP-32, *L. rhamnosus* bv-77, and *Bifidobacterium animalis* CP-9, plus diet and exercise guidance. A total of 82 children were enrolled, and 53 children completed the study.

**Results:**

The supplementation of multi-strain probiotics resulted in a significant effect demonstrating high-density lipoprotein (HDL) and adiponectin elevation. At the same time, body mass index (BMI) and serum total cholesterol, low-density lipoprotein (LDL), leptin, and tumor necrosis factor-alpha (TNF-α) levels were reduced. *Lactobacillus* spp. and *B. animalis* were particularly increased in subjects who received probiotic supplements. The abundance of *Lactobacillus* spp. was inversely correlated with the ether lipid metabolism pathway, while that of *B. animalis* was positively correlated with serum adiponectin levels.

**Conclusion:**

Our results show that obesity-related gut dysbiosis can be reshaped by the supplementation of a multi-strain probiotic to improve lipid metabolism. The regular administration of a multi-strain probiotic supplement may be helpful for weight control and health management in overweight and obese children.

## Introduction

According to the estimations of the World Health Organization (WHO), the prevalence of overweight or obese children and adolescents aged 5–19 years increased by more than four-fold, from 4 to 18%, globally from 1975 to 2016 (1). Compared to people with normal weight, obese people have a risk of diabetes, metabolic syndrome, and dyslipidemia that is 3 times higher compared to their normal-weight counterparts and a risk of hypertension, cardiovascular disease, knee arthritis, and gout that is 2 times higher (2). Childhood obesity has a number of negative health effects, such as early onset diabetes, high blood pressure, hyperlipidemia, and cardiovascular disease. Moreover, studies have found that obese children have a higher blood vessel stiffness, faster heartbeat, and poorer vascular endothelial function than normal-weight children (3). Long-term follow-up studies have revealed that the long-term effects of childhood obesity may cause abnormal heart structures and may result in a higher risk for cardiovascular disease in adults (4).

Although the main cause of obesity is excessive calorie intake and insufficient physical activity, recent studies have indicated that lack of sleep, poor eating habits, and the dysbiosis of intestinal bacteria can be attributed to obesity (5). Studies have further demonstrated that the composition of the intestinal flora in obese and normal-weight individuals is distinctly different. For instance, *Firmicutes* and *Staphylococcus aureus* were increased while *Bacteroidetes* and *Bifidobacterium* were decreased in obese children compared to lean children (6, 7). At present, it is not yet clear how the intestinal flora modulates obesity. Hypothetical mechanisms include changing the absorption, storage, and utilization of nutrients, and the regulation of energy metabolism and body inflammation (8).

According to the FAO/WHO, probiotics are “live microorganisms that, when administered in adequate amounts, confer a health benefit on the host” (9, 10). The most commonly used probiotic genera are *Bifidobacterium* and *Lactobacillus*. Numerous studies have revealed that obese adults who use probiotic-related products such as prebiotics, dairy products containing probiotics, and synbiotics or who take probiotic bacteria directly can effectively control their body weight (11). Nevertheless, it is critically noted that the effect of probiotics in obese children may not be the same as those observed in obese adults. Clinical investigations in this field remain very limited. In 2013, Safavi’s group reported the positive effect of a synbiotic product containing probiotics, which reduced the body weight, body mass index (BMI), and serum lipid levels in obese children (12). Contrastively, Jones’ group reported the null effect of a probiotic supplement (VSL#3^®^) intervention in obese Hispanic adolescents in 2018 (13). Therefore, there is a keen need to clarify specific probiotic strains or products that can provide positive effects on weight control in obese children. In our previous study, the weight-reducing effect of a multi-strain probiotic supplement was validated in high-fat diet (HFD)-induced obese rats (14). The multi-strain probiotic supplement contained three strains: *L. rhamnosus* bv-77, *B. lactis* CP-9, and *L. salivarius* AP-32. These three probiotic strains were able to reduce the ketone body level and alleviate body fat formation in rats when administered separately or together.

In this study, a clinical trial in overweight and obese children was carried out to explore whether an oral supplement of the three aforementioned multi-strain probiotics can help obese or overweight children to lose weight and improve metabolic disorders. In addition to weight control, the effect of this probiotic mixture was evaluated according to physiological observations of the subject, such as height, waist circumference, hip circumference, body fat, and blood pressure. Blood chemistry, including blood sugar, blood fat, liver function, and kidney functions were investigated. Serum cytokine and adipokine levels, such as those of TNF-α, leptin, and adiponectin were also analyzed. Eventually, next generation sequencing (NGS) was performed to reveal the influence of probiotic supplements on the gut microbiota.

## Materials and methods

### Ethics, informed consent, and permissions

This randomized, double-blind, placebo-controlled clinical study was carried out in the China Medical University Children’s Hospital. According to documentation from the Ministry of Health and Welfare, children with a BMI equal to or higher than the age- and sex-specific 85th percentile were overweight, and those with a BMI that was equal to or higher than the 95th percentile were obese. Overweight/obese children aged 6–18 years old were recruited through meetings with their parents. Children were excluded if they (1) had underlying conditions and other severe chronic diseases, (2) were on anti-obesity medication, (3) had already been taking probiotic products for a long time, and (4) were taking antibiotics. All of the qualified children whose parents had given informed consent were placed into randomized groups for the trial (*n* = 82). The study protocol (IRB No. CMUH105-REC2-096) was approved by the ethics committee of China Medical University Hospital. The trial is registered with the trial registry under code NCT03883191.

### Study design and subjects

The intervention lasted 3 months. Every day, the subjects in the study group took three packages of the supplement, which contained functional ingredients (i.e., white kidney bean extract: 300 mg, Psyllium husk: 100 mg, and Garcinia cambogia extract: 100 mg) and probiotics. The subjects were advised to take one package 30 min prior to each meal and to take three packages in total per day. Every package contained a total number of 10^10^ colony-forming units (CFU) including *Lactobacillus salivarius* AP-32 (10^9^ CFU), *Lactobacillus rhamnosus* bv-77 (10^9^ CFU), and *Bifidobacterium animalis* CP-9 (8 × 10^9^ CFU). *L. salivarius* AP-32 was isolated from a healthy human gut and deposited as BCRC910437 and CCTCC M 2011127. *L. rhamnosus* bv-77 was isolated from human breast milk and deposited as BCRC 910533 and CCTCC M 2014589. *B. animalis* CP-9 was isolated from human breast milk and deposited as BCRC 910645 and CCTCC M 2014588. At the same time, the subjects in the placebo group took three packages of the supplement containing the same ingredients without any of the probiotics. The doctors, parents, children, and investigators were unaware of which set of packages contained the probiotic mixtures until the end of the intervention and after the analysis was performed. During the intervention, other products containing probiotics were forbidden.

**FIGURE 1:**
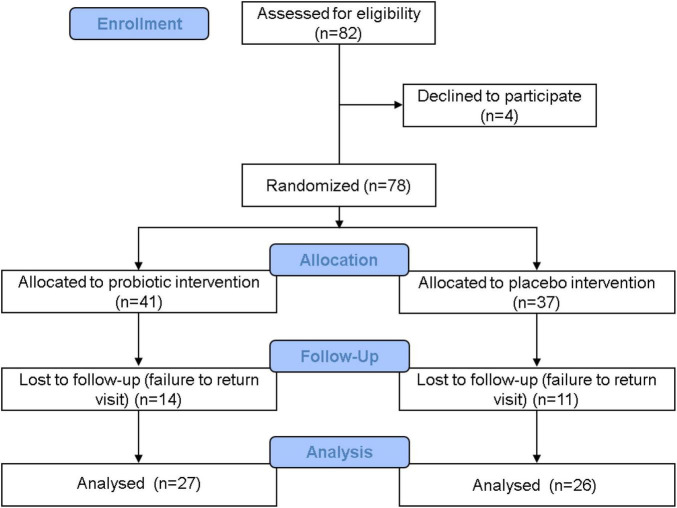
Clinical trial flowchart. A total of 82 people were admitted in the recruitment stage, and 4 people withdrew before the next stage. A total of 78 people entered the distribution stage: 41 people were allocated to the probiotic group, and 37 people were allocated to the placebo group. During the trial period, 14 people from the probiotic group withdrew, and 11 people from the placebo group withdrew. In the end, 27 people in the probiotic group and 26 people in the placebo group completed the study. Data from these 53 people were accounted for in the statistical analysis.

A detailed participant flowchart showing the baseline visit to the end visit is shown in Figure 1. Subjects visited Dr. An-Chyi Chen at the special clinic for pediatric obesity at the China Medical University Children’s Hospital. Information on the child’s family, living environment, child’s nutrition habits, exercise habits, and illnesses was collected. The child’s body height, body weight, blood pressure, heart rate, waistline, and hip circumference were also recorded. Body fat was measured at four sites: biceps, triceps, lower scapula, and thighs using Baseline Skinfold Calipers (Fabrication Enterprises, Inc., Pakistan). Symptoms such as acanthosis nigricans, obesity lines, gynecomastia, and snoring were diagnosed by the doctor. Fecal samples were collected at the beginning and the end of the study.

### Blood biochemical analysis

#### Blood collection and examination

Blood samples were collected from the subjects at the Medicine Laboratory Department of China Medical University Hospital. Serum indicators for thyroxine (TSH, T3, and T4), blood glucose (fasting blood glucose, HbA1c, C-peptide, and insulin), blood lipids [total cholesterol, low-density lipoprotein (LDL), high-density lipoprotein (HDL), and triglycerides], liver function (GOT and GPT), and renal function (uric acid) were analyzed. Values were compared, and change rates were investigated before and after the 3-month intervention.

#### Enzyme-linked immunosorbent assay

The blood serum was obtained by centrifugation and analyzed by commercial enzyme-linked immunosorbent assay (ELISA) kits. The ELISA kits for the human inflammatory cytokine TNF-α were purchased from BioLegend, Inc., United States, and those for human leptin and adiponectin were purchased from R&D Systems, Inc., United States and LifeSpan BioSciences Inc., United States, respectively. All samples are tested in at least three replicates.

#### Statistical analysis

Due to the potential dispersion of data, the value of the continuous variable is expressed as the mean ± standard error deviation (SEM). Wilcoxon signed-rank test and Mann–Whitney U test was used to compare the differences within or between groups; the variable is expressed as an N value or percentage was analyzed by Fisher’s exact test. The percentage of the test item was presented as the standardized average percentage (Endpoint/Baseline). The heat map demonstrating the fecal microbial richness was produced by using Graphpad prism 8 (Graphpad Software, San Diego, CA, United States). SPSS 18 (IBM, United States) was used for statistical analysis, and *p* < 0.05 were considered to be statistically significant.

### Gut microbiota analysis

#### Fecal DNA extraction

Bacteria DNA was extracted from the fecal samples using the QIAamp Fast DNA Stool Mini Kit(Qiagen, Germany) with some modified instructions. Briefly, the stool sample was centrifuged at 13,200 rpm for 10 min to remove the storage buffer and lysis using InhibitEX buffer. After homogenization, proteinase K and ethanol were added to obtain the processed supernatant. Finally, the supernatant was washed with a QIAamp spin column and eluted with elution buffer. The concentration was assessed by NanoDrop 2000 and a 10× dilution was then performed with elution buffer.

#### Next generation sequencing (NGS) analysis

The gut microbiome library was constructed with the standard V3–V4 region of the 16S rRNA gene. PCR was amplified with KAPA HiFi hotstart readymix (Roche, United States) and purified with AMPure XP magnetic beads (Beckman Coulter, United States). The amplified and quality of the PCR product was assessed using a Fragment Analyzer (Advanced Analytical, United States) and was quantified using a Qubit 3.0 Fluorometer. Then, the library was sequenced on a MiSeq (Illumina, United States) with paired-end reads (2 × 301 nt) and at least 100,000 reads of every sample.

#### Bioinformatics Analysis and Statistics

The raw paired-end reads were trimmed, and those that passed the quality filters were assigned to operational taxonomic units (OTU) with ≥ 97% similarity according to the GreenGene Database (v13.8). OTU taxonomic, alpha diversity, beta diversity, and heatmaps were performed with MicrobiomeAnalyst,^1^ GraphPad Prism 8 (GraphPad Software, United States), and the CLC genomics workbench (Qiagen, Germany). The bacterial abundance analysis (Linear discriminant analysis Effect Size, LEfSe), which is widely used to analyze significant differences between groups, and functional analysis (Phylogenetic Investigation of Communities by Reconstruction of Unobserved States, PICRUSt) were performed using the Galaxy/Hutlab website.^2^ A *p*-value less than 0.05 was considered statistically significant. The statistical significance was further adjusted by False Discovery Rate (FDR). The FDR was performed by using the smallest Benjamini-Hochberg adjusted *p*-value when utilizing unpaired *t*-tests with a Welch’s correction. An FDR-adjusted *p*-value (also called *q*-value) of 0.05 indicates that 5% of significant tests may result in false positives.

## Results

The multi-strain probiotic blend reduced triacylglycerol (TG) accumulation in Caco-2 cells (Supplementary Figure 1) and was packaged as powder supplements. Children aged 6–18 years who met the eligibility criteria of overweight or obesity were recruited, and the trial was carried out as shown in the flowchart in Figure 1. A total of 82 people entered the distribution stage, and 53 people fully completed the trial. Data from 27 people in the probiotic group and from 26 people in the placebo group were analyzed and compared at the end of the trial. The baseline characteristics of every subject were recorded, and no significant differences were found between the placebo and probiotic groups, with the exception of systolic blood pressure (Supplementary Table 1).

### Probiotic supplements reduced body mass index and body weight in obese children

After a 3-month intervention, the BMI of both groups was significantly reduced, which was probably due to regular exercise and diet. The subjects were educated regarding their diet and received exercise guidance, but no fixed meal recipes were assigned, nor was there a mandatory exercise schedule in this trial. Importantly, the reduction in the BMI level was significantly greater in the probiotic group (Table 1). The BMI level reduced by 0.5 kg/m^2^ in the placebo group (*p = 0.015) and by 1.2 kg/m^2^ in the probiotic group (^***^p < 0.001), respectively. In addition, the BMI change rate was significantly different between the probiotic and placebo groups (^#^*p* = 0.026). As expected for children who are around age 11, there was a significant increase in body height in both groups (Table 1). Body height increased by 1.2 cm in the placebo group (^***^p < 0.001) and increased by 1.5 cm in the probiotic group (^***^p < 0.001). Body weight was reduced in both groups, but a significant reduction was only observed in the probiotic group. There was a 0.5 kg body weight reduction in the placebo group (*p* = 0.594) and a significant reduction of 1.5 kg in the probiotic group (^**^p = 0.007).

**TABLE 1:** The comparison of physiological and blood biochemical values obtained before and after the intervention.

Parameter	Probiotics	Placebo	*P-value* ^#^
**BMI** **(kg/m^2^)**			
Baseline	29.7 ± 1.1	29.7 ± 1.0	0.790
End	28.5 ± 1.1	29.2 ± 1.0	0.533
*P*-value*	< 0.001***	0.015*	
Change rate (%)	95.9 ± 0.8	98.1 ± 0.9	0.026^#^
**Weight (kg)**			
Baseline	68.2 ± 4.4	66.5 ± 4.6	0.824
End	66.7 ± 4.2	66.0 ± 4.6	> 0.999
*p*-value*	0.007**	0.594	
Change rate (%)	98.0 ± 0.7	99.6 ± 0.8	0.062
**Height (cm)**			
Baseline	149.8 ± 2.8	147.3 ± 3.1	0.533
End	151.3 ± 2.7	148.5 ± 3.0	0.522
*p*-value*	< 0.001***	< 0.001***	
Change rate (%)	101.1 ± 0.3	100.9 ± 0.2	0.915
**Waist circumference (cm)**			
Baseline	89.9 ± 2.7	90.4 ± 2.5	0.715
End	89.3 ± 2.8	89.7 ± 2.5	0.625
*p*-value*	0.228	0.061	
Change rate (%)	99.3 ± 0.7	99.3 ± 0.5	0.742
**Hip circumference (cm)**			
Baseline	98.0 ± 2.6	97.5 ± 2.6	0.873
End	96.1 ± 2.6	96.4 ± 2.7	0.845
*p*-value*	0.055	0.051	
Change rate (%)	98.2 ± 0.8	98.9 ± 0.6	0.669
**Systolic blood pressure (mmHg)**			
Baseline	113.8 ± 3.1	124.7 ± 3.4	0.031^#^
End	115.9 ± 3.5	123.5 ± 3.1	0.157
*p*-value*	0.605	0.819	
Change rate (%)	102.4 ± 2.4	100.5 ± 3.2	0.522
**Diastolic blood pressure (mmHg)**			
Baseline	72.9 ± 2.8	76.4 ± 2.7	0.277
End	68.3 ± 2.7	72.8 ± 2.9	0.306
*p*-value*	0.109	0.451	
Change rate (%)	96.3 ± 4.6	98.4 ± 5.4	0.637
**Heart rate (beats/min)**			
Baseline	88.9 ± 2.6	87.3 ± 2.7	0.986
End	89.2 ± 2.1	84.4 ± 2.6	0.383
*p*-value*	0.958	0.353	
Change rate (%)	101.9 ± 3.0	98.1 ± 3.3	0.708
**Glucose AC (mg/dl)**			
Baseline	89.3 ± 1.2	89.7 ± 1.4	0.215
End	88.9 ± 1.4	90.2 ± 1.5	0.393
*p*-value*	0.178	0.849	
Change rate (%)	99.7 ± 1.6	100.8 ± 1.7	0.488
**HbA1c (%)**			
Baseline	5.6 ± 0.1	5.6 ± 0.0	0.548
End	5.5 ± 0.1	5.6 ± 0.0	0.100
*p*-value*	0.531	0.055	
Change rate (%)	99.0 ± 1.2	101.1 ± 0.5	0.378
**C-peptide (ng/ml)**			
Baseline	2.1 ± 0.2	2.4 ± 0.2	0.255
End	2.0 ± 0.2	2.2 ± 0.2	0.203
*p*-value*	0.923	0.909	
Change rate (%)	100.7 ± 7.5	99.4 ± 6.0	0.901
**Insulin** **(μ IU/ml)**			
Baseline	13.8 ± 1.2	15.9 ± 2.0	0.887
End	15.9 ± 2.2	14.2 ± 1.4	0.972
*p*-value*	0.486	0.485	
Change rate (%)	114.4 ± 9.3	112.2 ± 13.9	0.423
**HOMA-IR**			
Baseline	3.1 ± 0.3	3.5 ± 0.4	0.838
End	3.5 ± 0.5	3.2 ± 0.3	0.972
*p*-value*	0.548	0.424	
Change rate (%)	115.8 ± 11.0	116.0 ± 16.0	0.374

Data are presented as mean ± SEM of the results from each subject. Change rate: value_end_/value_baseline_ × 100% in the same subject. *The Wilcoxon signed rank test was used to compare the difference between before and after the intervention in each group, *p < 0.05, **p < 0.01, and ***p < 0.001. ^#^The Mann–Whitney U test was used to compare the difference between placebo and probiotic groups, ^#^p < 0.05.

The number of people with acanthosis nigricans, striae, gynecomastia, and snoring was not affected by the intervention in either the placebo or the probiotic group (Supplementary Table 2). It should be noted that obesity is frequently associated with liver damage and elevated liver enzymes in serum (15). The probiotic supplement significantly reduced serum GOT and GPT (Supplementary Table 2). The additional physiological test values that were obtained before and after the intervention are compared in Table 1. The waist and hip circumference were slightly reduced in both groups, but the change rates did not reach a significant difference between the two groups. A significant difference was observed in the systolic blood pressure between the placebo and probiotic groups before the intervention, and this difference no longer existed after the intervention. The diastolic blood pressure and heartbeat were not affected in both groups. It is well known that obesity is highly associated with insulin sensitivity, so carbohydrate metabolism was also investigated. Neither the placebo nor probiotic interventions affected the serum levels of glucose AC, HbA1c, C-peptide, insulin, and HOMA-IR.

### Probiotic supplements modulated blood lipid content and increased serum adiponectin levels in obese children

**FIGURE 2:**
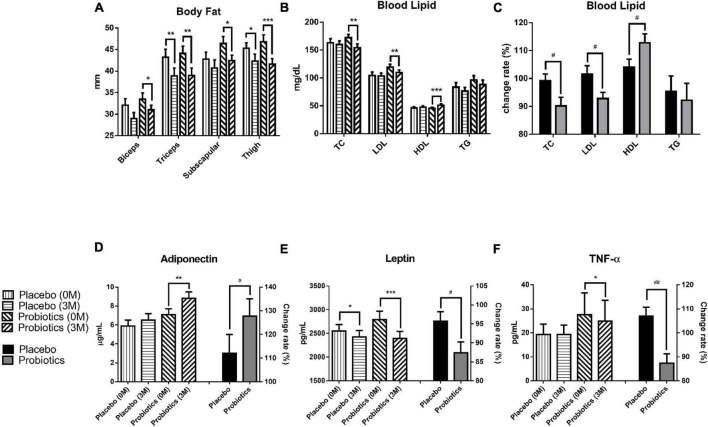
Probiotic supplements modulated blood lipids and adipokines in obese children. **(A)** The thickness of the subcutaneous tissue was measured at the biceps, triceps, subscapular, and thigh before (0 M) and after (3 M) the intervention. **(B)** TC (total cholesterol), LDL (low-density lipoprotein), HDL (high-density lipoprotein), and TG (triacylglycerol) were measured before and after the intervention. **(C)** The change rates of the TC, LDL, HDL, and TG were compared between the placebo and probiotic groups. **(D)** The adiponectin values before (0 M) and after (3 M) the intervention are plotted on the left *Y* axis, and the serum adiponectin change rate in the placebo and probiotic groups is plotted on the right *Y* axis. **(E)** The serum leptin values before and after the intervention are plotted on the left *Y* axis, and the change rate of leptin in the placebo and probiotic groups is plotted on the right *Y* axis. **(F)** The serum TNF-α values before and after the intervention are plotted on the left *Y* axis and the serum TNF-α change rate in the placebo and probiotic groups is plotted on the right *Y* axis. Data are presented as mean ± SEM. The Wilcoxon signed rank test was used to compare the differences between before and after the intervention within the group, *p < 0.05, **p < 0.01, and ***p < 0.001. The Mann–Whitney U test was used to compare the differences between the placebo and probiotic groups: ^#^*p* < 0.05 and ^##^*p* < 0.01.

After the 3-month intervention, the body fat was analyzed by measuring the thickness of the subcutaneous tissue at four sites: biceps, triceps, subscapular, and thigh. The result showed a significant body fat reduction at two sites in the placebo group, and at all sites in the probiotic group (Figure 2A). In the placebo group, body fat reduced from 43.2 ± 1.9 to 38.9 ± 1.8 mm (^**^p = 0.001) at the triceps and from 45.3 ± 1.3 to 42.3 ± 1.6 mm (*p = 0.032) at the thigh. In the probiotic group, body fat reduced from 33.5 ± 1.4 to 31.0 ± 1.1 mm (*p = 0.045), 44.1 ± 1.7 to 39.0 ± 1.7 mm (^**^p = 0.001), 46.4 ± 1.6 to 42.4 ± 1.3 mm (*p = 0.012), and from 46.8 ± 1.6 to 41.6 ± 1.3 mm (^***^p < 0.001) at the biceps, triceps, subscapular, and thigh, respectively.

The blood lipid content was significantly modulated in the probiotic group (Figures 2B,C). The total cholesterol (TC) level was reduced from 172.3 ± 5.9 to 154.3 ± 6.8 mg/dl in the probiotic group (^**^p = 0.004), and the change rate in the TC was significantly different between the probiotic and placebo groups (^#^*p* = 0.046). Moreover, two key components of TC, LDL and HDL, were investigated (Figures 2B,C). The LDL level was reduced from 119.4 ± 5.0 to 109.8 ± 4.4 mg/dl in the probiotic group (^**^p = 0.002), and the change rate in the LDL was significantly different between the probiotic and placebo groups (^#^*p* = 0.048). The HDL level increased from 45.6 ± 1.7 to 51.3 ± 2.3 mg/dl in the probiotic group (^***^p < 0.001), and the change rate in the HDL was also significantly different between the probiotic and placebo groups (^#^*p* = 0.039). There were a slight reduction in the triacylglycerol (TG) levels in both groups, and the difference did not reach statistical significance.

Adiponectin and leptin are cytokines that are excessively produced by adipocytes. Leptin is thought to be responsible for several cardiovascular diseases associated with obesity, while adiponectin is considered to be cardioprotective (16). The serum adiponectin level was increased from 7.1 ± 0.6 to 8.8 ± 0.7 μg/ml in the probiotic group (^**^p = 0.001), and the serum adiponectin change rate was significantly different between the probiotic and placebo groups (Figure 2D, ^#^*p* = 0.042). The serum leptin level was reduced from 2,552.2 ± 131.9 to 2,424.1 ± 135.1 pg/ml in the placebo group (*p = 0.040) and was reduced from 2,792.4 ± 175.1 to 2,393.3 ± 150.8 pg/ml in the probiotic group (Figure 2E, ^***^p < 0.001). Notably, serum leptin was reduced in both groups, but the serum leptin change rate remained significantly different between the probiotic and placebo groups (^#^*p* = 0.048).

Obesity is associated with chronic low-grade inflammation immune conditions and is usually accompanied by elevated pro-inflammatory cytokine levels such as TNF-α (17). Interestingly, the TNF-α level was not affected in the placebo group but was significantly reduced from 27.5 ± 9.2 to 24.9 ± 8.7 pg/ml in the probiotic group (Figure 2F, *p = 0.015). The TNF-α change rate was significantly different between the probiotic and placebo groups (^##^*p* = 0.001).

### Probiotic supplements changed the composition of the top 10 most abundant genera in obese children

**FIGURE 3:**
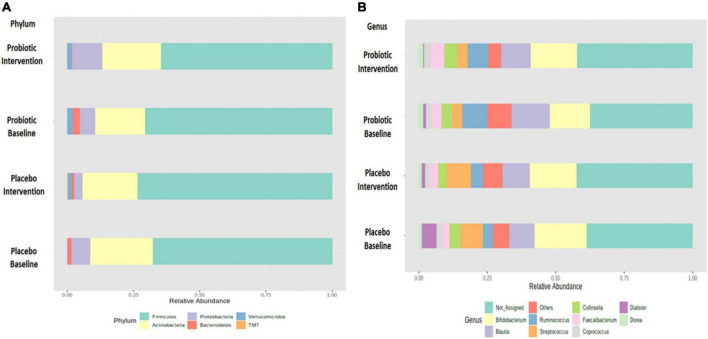
Probiotic supplements changed the composition of top 10 most abundant phyla and top 10 most abundant genera in obese children. The change in the microbial composition is presented at the **(A)** phylum and **(B)** genus levels.

Growing evidence has linked gut dysbiosis as a potential risk factor for the pathophysiology of obesity (18). To analyze whether the probiotic supplement would change the diversity of the gut microbiome, the alpha diversity (the complexity within a community) and beta diversity (the differences between microbial communities) of the fecal samples were further investigated. No significant changes were observed in neither the alpha nor the beta diversity between the probiotic and placebo groups (Supplementary Figure 2). The microbial compositions of the top 10 most abundant gut bacteria were compared in obese and overweight children before and after the intervention (Figure 3 and Supplementary Table 3). At the phylum level, *Proteobacteria* spp. increased (*P* = 0.042; FDR adjusted *q* = 0.255) and *Bacteroidetes* spp. decreased (*P* = 0.045; FDR adjusted *q* = 0.135) after the administration of the probiotic supplement (Figure 3A and Supplementary Figure 3A). At the genus level, *Blautia* spp. (*p* = 0.043; FDR adjusted *q* = 0.428) and *Ruminoccus* spp. (*p* = 0.049; FDR adjusted *q* = 0.162) decreased in the probiotic group compared to the placebo group (Figure 3B and Supplementary Figure 3B). Instead, the abundance of *Collinsella* spp. increased (*p* = 0.045; FDR adjusted *q* = 0.226) after the administration of the probiotic supplement.

### Gut microbiota were modulated differently in placebo and probiotic groups

**FIGURE 4:**
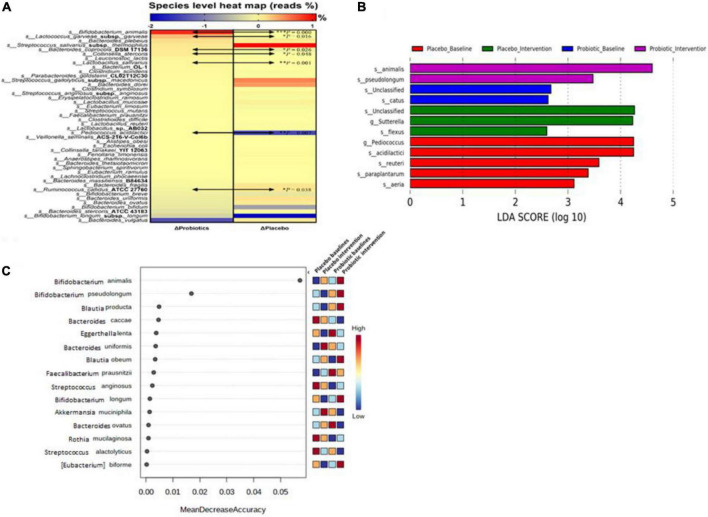
Probiotic supplements modulated fecal microbiota in obese children. **(A)** Microbial composition changes at the species level were presented as a heat map. **(B)** Linear discriminant analysis (LDA) Effect Size (LEfSe) analysis was performed in the placebo and probiotic groups before and after the intervention. **(C)** The importance of bacterial species in response to the intervention was ranked by Random forester analysis. Bacterial names are listed from top to down according to their change rates in the probiotic group. Data are presented as the mean values of the end-to-baseline ratio. The increase in the ratio is presented in red, and the decrease in the ratio is presented in blue. The Mann–Whitney U test was used to compare the continuous variables. Statistical comparisons were obtained by Student’s *t*-test, *p < 0.05, **p < 0.01, and ***p < 0.001.

Forty-four bacterial species were selected based on their abundance in the intestinal microbiota or according to their correlation to obesity as reported in the literature (19). In the probiotic group, *B. animalis* increased by 0.89% and *Bacteroides vulgatus* decreased by 1.37% after the intervention. In the placebo group, *Streptococcus salivarius* subsp. *thermophilus* increased by 1.07% and *B. longum* subsp. *longum* decreased by 2.00% (Figure 4A). Significant differences were observed in the change rates of seven species: *B. animalis*, *Lactococcus garvieae* subsp. *garvieae*, *Bacteroides coprocola* DSM 17136, *Collinsella stercoris*, *Lactobacillus salivarius*, *Pediococcus acidilactici*, and *Ruminococcus callidus* ATCC 27760, between probiotic and placebo groups (*p* < 0.001, 0.016, 0.026, 0.048, 0.001, 0.007, and 0.038, respectively).

To identify the core gut microbiota affected by the intervention, LEfSe [Linear discriminant analysis (LDA) Effect Size] was performed to analyze the core bacteria of the gut microbiome in obese and overweight children (Figure 4B). *B. animalis* and *B. pseudolongum* were significantly increased in the probiotic group. In contrast, *Sutterella* spp. and *Bacillus flexus* were significantly decreased in the probiotic group. Another online tool (Random forester analysis, Microbiome Analyst) was used to analyze the importance of the abundant bacteria from each group that was involved. *Bifidobacterium animalis* was found to be the most important abundant bacterium upon probiotic intervention (Figure 4C).

### The probiotic supplement increased *Lactobacillus* spp. and *B. animalis* in obese children

**FIGURE 5:**
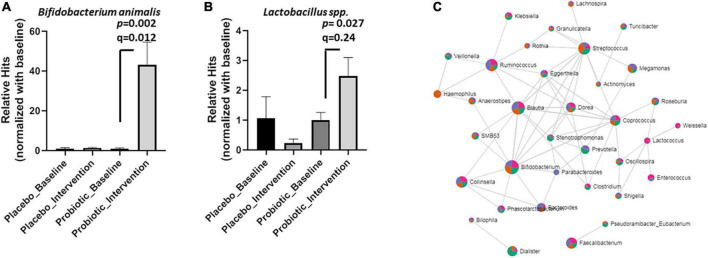
Probiotic supplements increased *Lactobacillus* spp. and *B. animalis* in obese children. The relative hits of **(A)** *B. animalis*, **(B)** *Lactobacillus* spp. were compared before and after the intervention in the placebo and probiotic groups. **(C)** The correlations between different bacteria in response to the intervention were displayed as a network. Data are presented as mean ± SEM. Statistical comparisons were obtained by Student’s *t*-test.

Based on the microbial compositions of the OTU (Figure 3) and the core bacteria found by the LEfSe and Random forester analysis (Figure 4), 11 genera and five species were commonly abundant between the placebo and probiotic groups (Supplementary Table 4). At the genus level, *Blautia* spp., *Ruminoccus* spp., *Streptococcus* spp. *Coprococcus* spp., *Dorea* spp., and *Bacteroides spp.* were decreased in the probiotic group, but *Streptococcus spp. Coprococcus spp., Dorea spp., and Bacteroides spp.* did not reach statistical significance (Supplementary Figure 3). Notably, *B. animalis* significantly increased in the probiotic group (*p* = 0.002; FDR adjusted *q* = 0.012, Figure 5A). However, the elevation of *Lactobacillus* spp. was less certain (*p* = 0.027; FDR adjusted *q* = 0.240, Figure 5B). Consequently, the correlation between different bacteria in response to the intervention was performed by an online tool [Correlation Analysis (SparCC), Microbiome Analyst] (Figure 5C). A gut microbiome network was created to display the complicated correlations between different bacterial genera. *Bifidobacterium* spp. played a central role in the network, which was in good agreement with the core bacteria deduced from the OTU, heatmap analysis, and LEfSe analysis.

### *Lactobacillus* spp. and *B. animalis* affected fat metabolism in different aspects

**FIGURE 6:**
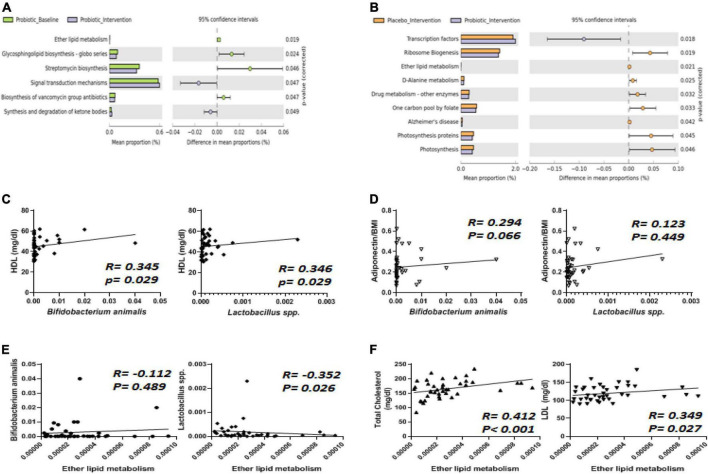
The abundance of *Bifidobacterium animalis* and *Lactobacillus* spp. displayed correlations with ether lipid metabolism, HDL, and adiponectin. **(A)** Six functional pathways were significantly affected by the intervention in the probiotic group. **(B)** Nine functional pathways were altered differently between the placebo and probiotic groups. The abundance of *B. animalis* and *Lactobacillus* spp. were correlated with **(C)** HDL, **(D)** adiponectin, and **(E)** ether lipid metabolism. **(F)** Total cholesterol (TC) and LDL were correlated with ether lipid metabolism.

The probiotic supplement was able to reshape obesity-related dysbiosis, and these alterations were able to be linked to the modulation of functional pathways *via* the Phylogenetic Investigation of Communities by Reconstruction of Unobserved States (PICRUSt) method. In the probiotic group, the intervention significantly decreased ether lipid metabolism compared to the probiotic baseline group (Figure 6A). Similarly, compared to the placebo group, the reduction in ether lipid metabolism was mainly attributed to probiotic supplementation (Figure 6B). It was noted that the probiotic supplement contained two *Lactobacillus* strains (*L. salivarius* AP-32 and *L. rhamnosus* bv-77) and one *Bifidobacterium* strain (*B. animalis* CP-9). Therefore, how *Lactobacillus* spp. and *B. animalis* are correlated with lipid metabolism were further investigated.

Spearman’s correlation analysis was performed to analyze how *Lactobacillus* spp. and *B. animalis* were correlated with all of the blood biochemistry values in obese children (Figures 6C–F). Among the blood lipid contents, HDL displayed a positive correlation with both *B. animalis* and *Lactobacillus* spp. (Figure 6C, both *p* = 0.029). One of the adipokines, adiponectin, displayed a positive correlation with *B. animalis* (Figure 6D). Ether lipid metabolism was negatively correlated to the abundance of *Lactobacillus* spp. (Figure 6E, *p* = 0.026). How ether lipid metabolism was correlated with blood lipids was also analyzed, and the results showed that TC and LDL were positively correlated with ether lipid metabolism (Figure 6F, *p* < 0.001 and 0.027, respectively).

## Discussion

It has been suggested that obese adults who use probiotic-related products can effectively control their weight and metabolic disorders related to weight (11). However, the effect of probiotics in obese children may not be the same as in adults (12, 13). In the present study, the supplementation of multi-strain probiotics, *L. salivarius* AP-32, *L. rhamnosus* bv-77, and *B. animalis* CP-9, significantly decreased BMI, TC, LDL, leptin, and TNF-α in overweight/obese children. Meanwhile, the intervention significantly increased serum HDL and adiponectin levels as well as reshaped or improved obesity-related gut dysbiosis. From the observation of the BMI profiles, the results indicate that the supplementation of functional ingredients together with probiotics was able to provide better weight control than supplementation with functional ingredients only. The BMI reduction in the placebo group was due to an increase in body height but not a decrease in body weight. The BMI reduction in the probiotic group was due to a simultaneous increase in body height and decrease in body weight. According to WHO guidelines, children should have at least 60 min of moderate-to-vigorous physical activity (PA) every day (20). However, a general survey reported that the amount of PA in grade 1 to grade 12 students in Taiwan did not meet WHO’s recommendation (21). In other words, exceeding the amount of PA recommended by the WHO can be more challenging for overweight/obese children and their families. In this trial, families were educated on the importance of PA, but it was not mandatory. An animal study showed that a combination of probiotics and exercise may synergistically assist obesity management and health improvement (22). Therefore, further studies combining programmed exercise with increased strength are needed to elucidate the synergistic effect of probiotics and PA in overweight/obese children.

Notably, better effects were observed in terms of body fat and blood lipids, such as reductions in the TC and LDL, and increase in the HDL were observed in the probiotic group. In the placebo group, body fat was reduced at two of the four measured sites, and no effects on blood lipids were observed. The placebo supplement contained functional ingredients, including white kidney bean extract, Psyllium husk, and Garcinia cambogia extract, which have been reported to modulate the gut microbiota in animals and humans (23–25). Therefore, the effect on weight loss could be expected in the placebo group (26–28). Our results indicated that the composition of the gut microbiota was altered differently in the placebo (functional ingredients) and probiotic (functional ingredients + probiotics) groups. Interestingly, the abundance of *B. animalis* was not affected in the placebo group, while it was significantly increased in the probiotic group. A high proportion (80%, 8 × 10^9^ CFU) of *B. animalis* CP-9 and a lower proportion of *Lactobacillus* (10%, 10^9^ CFU *L. salivarius* AP-32 and 10%, 10^9^ CFU *L. rhamnosus* bv-77) were intentionally used to compensate obesity-related dysbiosis (29) in this trial. Therefore, the ratio (AP-32: bv-77: CP-9 = 1: 1: 8) of probiotic strains in the supplement could be related to the relative abundance of the species in the fecal samples at the end of the trial. The probiotics were taken together with functional ingredients in the probiotic group, so it remains unclear to what extent probiotic bacteria itself could improve lipid metabolism in humans. One probiotic strain may reduce BMI without affecting lipid metabolism-related parameters (30). Moreover, other studies have shown that the same fermented products have controversial effects on the serum lipid level in humans (31, 32). A clinical study showed that the supplementation of probiotic strains and functional ingredients had a positive influence on blood lipoprotein profiles (33). Taken together, supplementation with solely abiotic ingredients or biotic strains may not have the best effect due to the lipid metabolism complex in the human body. The combination of probiotics and functional ingredients may exert synergistic effects and provide a more profound outcome than either one of them.

Adipokines and adipocytokines are bioactive products that are secreted by the adipose tissue and have become important biomarkers for metabolic disorders in the current decade (34, 35). The imbalance of decreased adiponectin plasma concentrations and increased leptin levels is closely related to obesity (36, 37). Additionally, leptin upregulates tumor necrosis factor alpha (TNF-α) expression (38). TNF-α is a multifunctional cytokine, and its elevated production leads to the inflammatory nature of obesity (39). Changing the efficiency of cellular fatty acid uptake to modulate leptin expression and production could be feasible (40). In a previous study, the same multi-strain supplement used in this study, which contained AP-32, bv-77, and CP-9 showed a better capability to reduce the ketone body, and non-esterified fatty acids (NEFA), and blood lipids than a mono-strain supplement in HFD-induced obese rodents (14). In this study, adiponectin upregulation and leptin and TNF-α downregulation were demonstrated in humans. Studies have reported that supplementation with *Bifidobacterium* spp. is able to induce adiponectin expression in animal models (41, 42). With the results of the *in vitro* TG accumulation assay, the supplement of this probiotic mixture may reduce lipid absorption in the small intestine and then reduce the lipid levels in blood. A lower serum lipid level may downregulate leptin secretion and then increase adiponectin levels. Leptin downregulation consequently resulted in downregulated TNF-α expression and ameliorated chronic inflammation. Adipose tissue inflammation increases the infiltration of M1 macrophage in the liver, and TNF-α is a potent pro-inflammatory mediator that is secreted by activated M1 macrophages (43). Obesity induces TNF-α elevation in the blood and liver (44). TNF-α elevation in the liver particularly influences the progression of non-alcoholic fatty liver disease (NAFLD) and results in liver damage (45). Higher incidences of abnormal GPT levels have been ascribed to the higher degree of obesity in children (46). In this study, the serum levels of GOT and GPT were responsive to treatment in both the placebo and probiotic groups. All of the participants received dietary fiber in their supplement, demonstrating the importance of fiber intake to liver health (47–49).

Ten years ago, the novel concept of “MicrObesity” (Microbes and Obesity) was proposed to address the specific role of dysbiosis and its impact on host metabolism and energy storage (50). Since then, more and more differences in the gut microbiota have been observed between obese and lean individuals. Decreased levels of the bacteria *Bifidobacterium*, *Desulfovibrio*, and *Lactobacillus* were reported to be associated with obesity in children (51), and high levels of *Bacteroides fragilis* (*B. fragilis*) and *Escherichia coli* (*E. coli*) were found in overweight/obese children (52). In this study, *Bifidobacterium* (CP-9) and *Lactobacillus* (bv-77 and AP-32) supplementation increased their populations in the gut and elevated the abundance of *Desulfovibrio*, which was not included in the probiotic package, unexpectedly. Although the role of *Desulfovibrio* in human health remains controversial and may be age-related (51), more studies are required to verify whether the effect of *Desulfovibrio* was species-specific (53). Moreover, lower levels of *B. fragilis* and *E. coli*, whose abundances were positively correlated with obesity, were observed in the probiotic group. In short, the probiotic intervention was able to modulate the gut microbiota and reshape dysbiosis in overweight/obese children.

Many studies have suggested that the function and efficacy of multi-strain probiotic supplements should be superior to mono-strain ones, demonstrating synergy among strains (54). The multi-strain probiotic supplement used in this trial contained three species from two genera. The effects of mono- (AP-32, bv-77, or CP-9) and multi-strain (AP-32/bv-77/CP-9) probiotic supplementations have been previously investigated in obese rats (14). Consistent with the animal study, improved blood lipid profiles and gut microbiota modulation was observed in the subjects of this trial who received multi-strain probiotics. In terms of the blood lipid profile, the improvement was indistinguishable among the animals receiving mono- and multi-strains over the course of 4 weeks. However, the amelioration of weight gain and the modulation of the gut microbiota were more effective in animals receiving multi-strain probiotics. For instance, the abundance of *B. animalis* was significantly increased by the multi-strain probiotics over the course of 4 weeks, but surprisingly, the increment achieved by the mono-strain supplement (i.e., *B. animalis* CP-9) was not significant due to wide variation in the results. We speculated that the multi-strain supplements containing *Lactobacillus spp.* (*L. salivarius* AP-32 and *L. rhamnosus* bv-77) created an optimal pH environment for probiotic colonization in the small intestine and promoted the better survival of the *B. animalis* subsp. *lactis* CP-9 in the large intestine. This hypothesis needs to be supported by further studies, such as *in vivo* bioluminescence imaging in whole animals after one oral administration (55). The mechanism of these additive effects is still far from clear and is believed to involve complicated networks of cell–cell interactions and communications known as quorum sensing (QS) (56). Although observations have found the association between the gut microbiota and metabolism, the evidence of strong linkages between specific bacteria and functional pathways remained rare. In our study, the analysis was focused on the correlation of the gut microbiota with lipid metabolism, and the results elucidated the role of *Lactobacillus* spp. and *B. animalis* in fat metabolism. *Lactobacillus* spp. was positively correlated with HDL and negatively correlated with TC and LDL. A meta-analysis revealed a significant reduction in TC using *L. plantarum* and a reduction in LDL-C using *L. plantarum* or *L. reuteri* (57). Two *Lactobacillus* strains, *L. salivarius* AP-32 and *L. rhamnosus* bv-77, were included in our multi-strain probiotic blend, and further studies will be needed to verify their specific roles in lipid profiling. Our results also showed a positive correlation between the abundance of *B. animalis* in the gut microbiota and serum HDL and adiponectin. Several beneficial effects on inflammatory and oxidative biomarkers were seen in healthy subjects and metabolic syndrome patients received *B. lactis* HN019 supplementation (58). In other words, our results indicate that *Lactobacillus* spp. had more of an impact on the blood lipid profile, while *B. animalis* had more of an impact on anti-inflammation. Our results reveal the delicate differences between different bacteria and provide insight into the synergy of probiotic strains in the host. Taken together, this clinical study demonstrated that a well-designed multi-strain probiotic supplement can exert synergistic effects and regulate metabolism from more comprehensive aspects.

Ether lipids are major polar lipids in the cell envelope and play potential roles in cellular functions, such as membrane homeostasis and membrane trafficking. Ether lipids can affect cholesterol homeostasis, and crosstalk between the two metabolic pathways was proposed (59). A group of ether-linked lipids has been reported to be elevated in morbidly obese humans (60). In this study, ether lipid metabolism was positively associated with total cholesterol and LDLs. Intriguingly, *Lactobacillus* spp. displayed a negative association with ether lipid metabolism. Elevated TC and LDL levels were associated with a higher risk of coronary heart disease, Alzheimer’s disease, and mild cognitive impairment (61, 62). Therefore, supplementation with the multi-strain probiotic blend elevated *Lactobacillus* spp. in the gut microbiota and potentially reduced the risk of dyslipidemia-related diseases.

Other than the ether lipid metabolism pathway, the correlation analysis indicated the induction of signal transduction *via* intervention in the probiotic group. Compared to the placebo groups, the functional pathway of the transduction factors was also significantly induced in the probiotic group. Signal transduction is a series of molecular events by which a chemical or physical signal is transmitted through a cell and ultimately results in a cellular response. During the signal transduction process, the activity of nuclear transcription factors is carefully modulated to exert a precise cellular response (63). Energy metabolism regulation requires the activation of the corresponding signal transduction pathways. For instance, extra-cellular insulin regulates sugar metabolism by activating the cascade of intra-cellular phosphatases and substrates (64). Therefore, part of signal transduction activation may be due to fat metabolism modulation. Notably, the probiotic supplement also affected other functional pathways, such as the biosynthesis of antibiotics and drug metabolism. Moreover, lower levels of *B. fragilis* and *E. coli* were observed in the probiotic group. Based on the results, further studies investigating the immune response pathways against pathogens are recommended.

There were some key limitations including diet and exercise which were not strictly recorded during the clinical trial. They are also confounding factors that may impact the composition of gut microbiota. For instance, the ratio of *Bacteroides* and *Prevotella* can be modulated by a diet high in animal protein and saturated fat or a plant-based diet rich in fiber and simple carbohydrates (65). Exercise is supposed to reduce inflammatory infiltration but increase microbial diversity in the gut (66). Our results showed that the abundance of *Bacteroides* was reduced in both groups, implicating that the subjects of both two groups might take a more plant-based diet according to the diet guidance and no significant impact of the diet on gut microbiota was observed between them. Although TNF-α was reduced in the probiotic group, gut microbial diversity was not affected in both groups. This result might indicate that the subjects of both groups might not take enough exercise to impact their gut microbial diversity. Further studies with well-designed diet and exercise courses will be warranted to demonstrate the real effectiveness of the multi-strain probiotics against obesity. In addition, functional ingredients contained 300 mg of white kidney bean extract, 100 mg of psyllium husk, and 100 mg of garcinia cambogia extract per package used in our study. Although these ingredients were reported to modulate gut microbiota (23–25), we could not clearly conclude their impacts on gut microbiota and weight control when in combination. The amounts used in this study might be not high enough to observe the impacts on weight control and on alterations of gut microbiota such as *Prevotella* and *Streptococcus* in mice as well as *Veillonella* and *Subdoligranulum* in humans (23–25). For example, a daily supplement of 7 g psyllium husk can increase *Veillonella* but decrease *Subdoligranulum* in healthy adults (67). On the other hand, although we evenly allocated participants according to their physical measures, the bias of different systolic blood pressures occurred between groups before the intervention. The higher systolic blood pressure may reflect a poor cardiovascular condition and a different type of microbiota dysbiosis from the probiotics group and thus may lead to a bias of the impacted alterations of gut microbiota in the placebo group when compared to the probiotic group. Although we did not observe significant differences in the baseline of several microbial biomarkers in our study (Supplementary Figure 3) between the two groups, the extension of the allocation criteria to cardiovascular measures will be necessary to avoid bias when including high BMI participants in further study. It is eventually noted the possibility of a pair of variables that show a significant *p*-value with no biological association due to aleatory phenomena when comparing the 16S rRNA sequencing. A stricter significance threshold for individual comparisons will be required. In our case, the elevation of *Bifidobacterium animalis* displayed a statistical significance with both *p*- and *q*-values (adjusted *p*-value by FDR) less than 0.05. However, in the case of *Lactobacillus* spp., a *p*-value less than 0.05 was presented by using a loose threshold, but a *q*-value represented higher than 0.05 using a strict threshold. Therefore, the former statistical significance can be included and interpreted.

In conclusion, the probiotic blend of *L. salivarius* AP-32, *L. rhamnosus* bv-77, and *B. animalis* CP-9 (1: 1: 8) enhanced the effect of functional ingredients and displayed a greater influence on improving lipid metabolism. The probiotic supplement was able to modulate the gut microbiota and consequently improve the blood lipid profile, alleviate low-grade inflammation, and reduce body weight in overweight/obese subjects. However, the dietary supplement demonstrated limitations in terms of weight loss, with BMI reductions of less than 5% after the 3-month intervention. Weight loss is not easy, and better outcomes can make it easier for people to maintain their motivation to lose weight. Future studies combining diet control and exercise programs are suggested to evaluate the facilitating effects of probiotic supplements on weight loss.

## Data availability statement

The original contributions presented in this study are included in the article/Supplementary Material, further inquiries can be directed to the corresponding authors.

## Ethics statement

The studies involving human participants were reviewed and approved by the CMUH105-REC2-096 – China Medical University Hospital. Written informed consent to participate in this study was provided by the participants or their legal guardian/next of kin. Written informed consent was obtained from the minor(s)’ legal guardian/next of kin for the publication of any potentially identifiable images or data included in this article.

## Author contributions

Y-TY and H-CL contributed equally to the conception and design of the study. A-CC and T-JF contributed equally to this work. A-CC, T-JF, H-HH, J-FC, Y-WK, and Y-YH organized the database. S-YT and S-FW performed the statistical analysis. A-CC and T-JF wrote the first draft of the manuscript. H-HH, J-FC, Y-WK, and Y-YH wrote sections of the manuscript. All authors contributed to the manuscript revision, read, and approved the submitted version.

## Footnotes

1
https://www.microbiomeanalyst.ca/MicrobiomeAnalyst/upload/OtuUploadView.xhtml

2
http://huttenhower.sph.harvard.edu/galaxy/

## References

[1] Ncd Risk Factor Collaboration [Ncd-RisC]. Worldwide trends in body-mass index, underweight, overweight, and obesity from 1975 to 2016: a pooled analysis of 2416 population-based measurement studies in 128.9 million children, adolescents, and adults. *Lancet.* (2017) 390:2627–42. 10.1016/S0140-6736(17)32129-3 29029897 PMC5735219

[2] Ginsberg HN MacCallum PR. The obesity, metabolic syndrome, and type 2 diabetes mellitus pandemic: part I. Increased cardiovascular disease risk and the importance of atherogenic dyslipidemia in persons with the metabolic syndrome and type 2 diabetes mellitus. *J Cardiometab Syndr.* (2009) 4:113–9. 10.1111/j.1559-4572.2008.00044.x 19614799 PMC2901596

[3] Nunez F Martinez-Costa C Sanchez-Zahonero J Morata J Chorro FJ Brines J. Carotid artery stiffness as an early marker of vascular lesions in children and adolescents with cardiovascular risk factors. *Rev Esp Cardiol.* (2010) 63:1253–60. 10.1016/s1885-5857(10)70250-4 21070721

[4] Field AE Cook NR Gillman MW. Weight status in childhood as a predictor of becoming overweight or hypertensive in early adulthood. *Obes Res.* (2005) 13:163–9. 15761176 10.1038/oby.2005.21 PMC1989681

[5] Sanchez M Panahi S Tremblay A. Childhood obesity: a role for gut microbiota? *Int J Environ Res Public Health.* (2014) 12:162–75. 25546278 10.3390/ijerph120100162 PMC4306855

[6] Bervoets L Van Hoorenbeeck K Kortleven I Van Noten C Hens N Vael C Differences in gut microbiota composition between obese and lean children: a cross-sectional study. *Gut Pathog.* (2013) 5:10. 23631345 10.1186/1757-4749-5-10 PMC3658928

[7] Kalliomaki M Collado MC Salminen S Isolauri E. Early differences in fecal microbiota composition in children may predict overweight. *Am J Clin Nutr.* (2008) 87:534–8. 10.1093/ajcn/87.3.534 18326589

[8] Turnbaugh PJ Gordon JI. The core gut microbiome, energy balance and obesity. *J Physiol.* (2009) 587(Pt 17):4153–8. 19491241 10.1113/jphysiol.2009.174136 PMC2754355

[9] Fao/Who. *Guidelines for the Evaluation of Probiotics in Food.* London: Food and Agriculture Organization of the United Nations/World Health Organization (2002).

[10] Hill C Guarner F Reid G Gibson GR Merenstein DJ Pot B Expert consensus document. The International scientific association for probiotics and prebiotics consensus statement on the scope and appropriate use of the term probiotic. *Nat Rev Gastroenterol Hepatol.* (2014) 11:506–14. 24912386 10.1038/nrgastro.2014.66

[11] Zhang Q Wu Y Fei X. Effect of probiotics on body weight and body-mass index: a systematic review and meta-analysis of randomized, controlled trials. *Int J Food Sci Nutr.* (2015) 67:571–80. 27149163 10.1080/09637486.2016.1181156

[12] Safavi M Farajian S Kelishadi R Mirlohi M Hashemipour M. The effects of synbiotic supplementation on some cardio-metabolic risk factors in overweight and obese children: a randomized triple-masked controlled trial. *Int J Food Sci Nutr.* (2013) 64:687–93. 10.3109/09637486.2013.775224 23477506

[13] Jones RB Alderete TL Martin AA Geary BA Hwang DH Palmer SL Probiotic supplementation increases obesity with no detectable effects on liver fat or gut microbiota in obese Hispanic adolescents: a 16-week, randomized, placebo-controlled trial. *Pediatr Obes.* (2018) 13:705–14. 29493105 10.1111/ijpo.12273 PMC6113106

[14] Liao C-A Huang C-H Ho H-H Chen J-F Kuo Y-W Lin J-H A combined supplement of probiotic strains AP-32, bv-77, and CP-9 Increased *Akkermansia mucinphila* and reduced non-esterified fatty acids and energy metabolism in HFD-induced obese rats. *Nutrients.* (2022) 14:527. 10.3390/nu14030527 35276886 PMC8839477

[15] Nakamura S Takezawa Y Nakajima Y Maeda T. Elevation of glutamic pyruvic transaminase and gamma-glutamyl transpeptidase in obesity. *Tohoku J Exp Med.* (1980) 132:473–8. 6114578 10.1620/tjem.132.473

[16] Abel ED Litwin SE Sweeney G. Cardiac remodeling in obesity. *Physiol Rev.* (2008) 88:389–419. 18391168 10.1152/physrev.00017.2007 PMC2915933

[17] Chollet-Martin S Fricker J Apfelbaum M Gougerot-Pocidalo MA. Tumor necrosis factor and obesity. *Ann Intern Med.* (1989) 110:666–7. 2930100 10.7326/0003-4819-110-8-666_2

[18] Backhed F Ding H Wang T Hooper LV Koh GY Nagy A The gut microbiota as an environmental factor that regulates fat storage. *Proc Natl Acad Sci USA.* (2004) 101:15718–23. 15505215 10.1073/pnas.0407076101 PMC524219

[19] Muscogiuri G Cantone E Cassarano S Tuccinardi D Barrea L Savastano S Gut microbiota: a new path to treat obesity. *Int J Obes Suppl.* (2019) 9:10–9. 31391921 10.1038/s41367-019-0011-7 PMC6683132

[20] Bull FC Al-Ansari SS Biddle S Borodulin K Buman MP Cardon G World Health Organization 2020 guidelines on physical activity and sedentary behaviour. *Br J Sports Med.* (2020) 54:1451–62. 33239350 10.1136/bjsports-2020-102955 PMC7719906

[21] Wu CL Chang CK. Results from the Chinese Taipei (Taiwan) 2018 report card on physical activity for children and youth. *J Exerc Sci Fit.* (2019) 17:8–13. 30662508 10.1016/j.jesf.2018.10.005 PMC6323190

[22] Hsu YJ Chiu CC Lee MC Huang WC. Combination of treadmill aerobic exercise with *Bifidobacterium longum* OLP-01 supplementation for treatment of high-fat diet-induced obese murine model. *Obes Facts.* (2021) 14:306–19. 10.1159/000516865 34077946 PMC8255637

[23] Wang S Guo C Xing Z Li M Yang H Zhang Y Dietary intervention with alpha-amylase inhibitor in white kidney beans added yogurt modulated gut microbiota to adjust blood glucose in mice. *Front Nutr.* (2021) 8:664976. 10.3389/fnut.2021.664976 34712684 PMC8545863

[24] Yang C Liu S Li H Bai X Shan S Gao P The effects of psyllium husk on gut microbiota composition and function in chronically constipated women of reproductive age using 16S rRNA gene sequencing analysis. *Aging (Albany NY).* (2021) 13:15366–83. 34081625 10.18632/aging.203095 PMC8221300

[25] Heo J Seo M Park H Lee WK Guan LL Yoon J Gut microbiota modulated by probiotics and *Garcinia* cambogia extract correlate with weight gain and adipocyte sizes in high fat-fed mice. *Sci Rep.* (2016) 6:33566. 10.1038/srep33566 27658722 PMC5034228

[26] Nolan R Shannon OM Robinson N Joel A Houghton D Malcomson FC. It’s no has bean: a review of the effects of white kidney bean extract on body composition and metabolic health. *Nutrients.* (2020) 12:1398. 10.3390/nu12051398 32414090 PMC7284421

[27] Moreno LA Tresaco B Bueno G Fleta J Rodriguez G Garagorri JM Psyllium fibre and the metabolic control of obese children and adolescents. *J Physiol Biochem.* (2003) 59:235–42. 10.1007/BF03179920 15000455

[28] Haber SL Awwad O Phillips A Park AE Pham TM. *Garcinia* cambogia for weight loss. *Am J Health Syst Pharm.* (2018) 75:17–22. 29317394 10.2146/ajhp160915

[29] Stojanov S Berlec A Strukelj B. The influence of probiotics on the firmicutes/bacteroidetes ratio in the treatment of obesity and inflammatory bowel disease. *Microorganisms.* (2020) 8:1715. 10.3390/microorganisms8111715 33139627 PMC7692443

[30] Kadooka Y Sato M Imaizumi K Ogawa A Ikuyama K Akai Y Regulation of abdominal adiposity by probiotics (*Lactobacillus gasseri* SBT2055) in adults with obese tendencies in a randomized controlled trial. *Eur J Clin Nutr.* (2010) 64:636–43. 10.1038/ejcn.2010.19 20216555

[31] St-Onge MP Farnworth ER Savard T Chabot D Mafu A Jones PJ. Kefir consumption does not alter plasma lipid levels or cholesterol fractional synthesis rates relative to milk in hyperlipidemic men: a randomized controlled trial [ISRCTN10820810]. *BMC Complement Altern Med.* (2002) 2:1. 10.1186/1472-6882-2-1 11825344 PMC65674

[32] Fathi Y Ghodrati N Zibaeenezhad MJ Faghih S. Kefir drink causes a significant yet similar improvement in serum lipid profile, compared with low-fat milk, in a dairy-rich diet in overweight or obese premenopausal women: a randomized controlled trial. *J Clin Lipidol.* (2017) 11:136–46. 28391880 10.1016/j.jacl.2016.10.016

[33] Kullisaar T Zilmer K Salum T Rehema A Zilmer M. The use of probiotic L. fermentum ME-3 containing Reg’Activ cholesterol supplement for 4 weeks has a positive influence on blood lipoprotein profiles and inflammatory cytokines: an open-label preliminary study. *Nutr J.* (2016) 15:93. 10.1186/s12937-016-0213-6 27793203 PMC5084312

[34] Conde J Scotece M Gomez R Lopez V Gomez-Reino JJ Lago F Adipokines: biofactors from white adipose tissue. A complex hub among inflammation, metabolism, and immunity. *Biofactors.* (2011) 37:413–20. 10.1002/biof.185 22038756

[35] Lehr S Hartwig S Sell H. Adipokines: a treasure trove for the discovery of biomarkers for metabolic disorders. *Proteomics Clin Appl.* (2012) 6:91–101. 10.1002/prca.201100052 22213627

[36] Lopez-Jaramillo P Gomez-Arbelaez D Lopez-Lopez J Lopez-Lopez C Martinez-Ortega J Gomez-Rodriguez A The role of leptin/adiponectin ratio in metabolic syndrome and diabetes. *Horm Mol Biol Clin Investig.* (2014) 18:37–45. 25389999 10.1515/hmbci-2013-0053

[37] Ghantous CM Azrak Z Hanache S Abou-Kheir W Zeidan A. Differential role of leptin and adiponectin in cardiovascular system. *Int J Endocrinol.* (2015) 2015:534320. 26064110 10.1155/2015/534320 PMC4433709

[38] Lee SM Choi HJ Oh CH Oh JW Han JS. Leptin increases TNF-alpha expression and production through phospholipase D1 in Raw 264.7 cells. *PLoS One.* (2014) 9:e102373. 10.1371/journal.pone.0102373 25047119 PMC4105621

[39] Sethi JK Hotamisligil GS. Metabolic messengers: tumour necrosis factor. *Nat Metab.* (2021) 3:1302–12. 34650277 10.1038/s42255-021-00470-z

[40] Hajri T Hall AM Jensen DR Pietka TA Drover VA Tao H CD36-facilitated fatty acid uptake inhibits leptin production and signaling in adipose tissue. *Diabetes.* (2007) 56:1872–80. 10.2337/db06-1699 17440173

[41] Le TK Hosaka T Le TT Nguyen TG Tran QB Le TH Oral administration of *Bifidobacterium* spp. improves insulin resistance, induces adiponectin, and prevents inflammatory adipokine expressions. *Biomed Res.* (2014) 35:303–10. 10.2220/biomedres.35.303 25355437

[42] Le TK Hosaka T Nguyen TT Kassu A Dang TO Tran HB *Bifidobacterium* species lower serum glucose, increase expressions of insulin signaling proteins, and improve adipokine profile in diabetic mice. *Biomed Res.* (2015) 36:63–70. 10.2220/biomedres.36.63 25749152

[43] Wu X Xu W Feng X He Y Liu X Gao Y TNF-a mediated inflammatory macrophage polarization contributes to the pathogenesis of steroid-induced osteonecrosis in mice. *Int J Immunopathol Pharmacol.* (2015) 28:351–61. 10.1177/0394632015593228 26197804

[44] Luo Y Lin H. Inflammation initiates a vicious cycle between obesity and nonalcoholic fatty liver disease. *Immun Inflamm Dis.* (2021) 9:59–73. 33332766 10.1002/iid3.391 PMC7860600

[45] Kakino S Ohki T Nakayama H Yuan X Otabe S Hashinaga T Pivotal role of TNF-alpha in the development and progression of nonalcoholic fatty liver disease in a murine model. *Horm Metab Res.* (2018) 50:80–7. 10.1055/s-0043-118666 28922680

[46] Das RK Nessa A. Blood pressure in different levels of BMI. *Mymensingh Med J.* (2013) 22:699–705. 24292299

[47] Chang FT Hu SH Wang RS. The effectiveness of dietary instruction in obese school children of southern Taiwan. *Kaohsiung J Med Sci.* (1998) 14:528–35. 9796195

[48] Lee H Lee IS Choue R. Obesity, inflammation and diet. *Pediatr Gastroenterol Hepatol Nutr.* (2013) 16:143–52. 24224147 10.5223/pghn.2013.16.3.143 PMC3819692

[49] Li MM Zhou Y Zuo L Nie D Li XA. Dietary fiber regulates intestinal flora and suppresses liver and systemic inflammation to alleviate liver fibrosis in mice. *Nutrition.* (2021) 81:110959. 10.1016/j.nut.2020.110959 33059126

[50] Cani PD Delzenne NM. The gut microbiome as therapeutic target. *Pharmacol Ther.* (2011) 130:202–12. 21295072 10.1016/j.pharmthera.2011.01.012

[51] Vinke PC El Aidy S van Dijk G. The role of supplemental complex dietary carbohydrates and gut microbiota in promoting cardiometabolic and immunological health in obesity: lessons from healthy non-obese individuals. *Front Nutr.* (2017) 4:34. 10.3389/fnut.2017.00034 28791292 PMC5523113

[52] Indiani C Rizzardi KF Castelo PM Ferraz LFC Darrieux M Parisotto TM. Childhood obesity and firmicutes/bacteroidetes ratio in the gut microbiota: a systematic review. *Child Obes.* (2018) 14:501–9. 10.1089/chi.2018.0040 30183336

[53] Meyer B Kuehl J Deutschbauer AM Price MN Arkin AP Stahl DA. Variation among *Desulfovibrio* species in electron transfer systems used for syntrophic growth. *J Bacteriol.* (2013) 195:990–1004. 10.1128/JB.01959-12 23264581 PMC3571329

[54] Timmerman HM Koning CJ Mulder L Rombouts FM Beynen AC. Monostrain, multistrain and multispecies probiotics–a comparison of functionality and efficacy. *Int J Food Microbiol.* (2004) 96:219–33. 10.1016/j.ijfoodmicro.2004.05.012 15454313

[55] Van Zyl WF Deane SM Dicks LMT. *In vivo* bioluminescence imaging of the spatial and temporal colonization of *Lactobacillus plantarum* 423 and enterococcus mundtii ST4SA in the intestinal tract of mice. *BMC Microbiol.* (2018) 18:171. 10.1186/s12866-018-1315-4 30376820 PMC6208077

[56] Kwoji ID Aiyegoro OA Okpeku M Adeleke MA. Multi-strain probiotics: synergy among isolates enhances biological activities. *Biology (Basel).* (2021) 10:322. 10.3390/biology10040322 33924344 PMC8070017

[57] Wu Y Zhang Q Ren Y Ruan Z. Effect of probiotic *Lactobacillus* on lipid profile: a systematic review and meta-analysis of randomized, controlled trials. *PLoS One.* (2017) 12:e0178868. 10.1371/journal.pone.0178868 28594860 PMC5464580

[58] Bernini LJ Simao ANC de Souza CHB Alfieri DF Segura LG Costa GN Effect of *Bifidobacterium lactis* HN019 on inflammatory markers and oxidative stress in subjects with and without the metabolic syndrome. *Br J Nutr.* (2018) 120:645–52. 10.1017/S0007114518001861 30058513

[59] Jimenez-Rojo N Riezman H. On the road to unraveling the molecular functions of ether lipids. *FEBS Lett.* (2019) 593:2378–89. 10.1002/1873-3468.13465 31166014

[60] Donovan EL Pettine SM Hickey MS Hamilton KL Miller BF. Lipidomic analysis of human plasma reveals ether-linked lipids that are elevated in morbidly obese humans compared to lean. *Diabetol Metab Syndr.* (2013) 5:24. 10.1186/1758-5996-5-24 23672807 PMC3663699

[61] Shabana, Shahid SU Sarwar S. The abnormal lipid profile in obesity and coronary heart disease (CHD) in Pakistani subjects. *Lipids Health Dis.* (2020) 19:73. 10.1186/s12944-020-01248-0 32290855 PMC7158030

[62] Liu Y Zhong X Shen J Jiao L Tong J Zhao W Elevated serum TC and LDL-C levels in Alzheimer’s disease and mild cognitive impairment: a meta-analysis study. *Brain Res.* (2020) 1727:146554. 10.1016/j.brainres.2019.146554 31765631

[63] Karin M Smeal T. Control of transcription factors by signal transduction pathways: the beginning of the end. *Trends Biochem Sci.* (1992) 17:418–22. 1455510 10.1016/0968-0004(92)90012-x

[64] Posner BI. Insulin signalling: the inside story. *Can J Diabetes.* (2017) 41:108–13. 10.1016/j.jcjd.2016.07.002 27614806 PMC5272803

[65] Wu GD Chen J Hoffmann C Bittinger K Chen YY Keilbaugh SA Linking long-term dietary patterns with gut microbial enterotypes. *Science.* (2011) 334:105–8. 21885731 10.1126/science.1208344 PMC3368382

[66] Monda V Villano I Messina A Valenzano A Esposito T Moscatelli F Exercise modifies the gut microbiota with positive health effects. *Oxid Med Cell Longev.* (2017) 2017:3831972. 28357027 10.1155/2017/3831972 PMC5357536

[67] Jalanka J Major G Murray K Singh G Nowak A Kurtz C The effect of psyllium husk on intestinal microbiota in constipated patients and healthy controls. *Int J Mol Sci.* (2019) 20:433. 10.3390/ijms20020433 30669509 PMC6358997

