# Supplementary material for: A multi-strain probiotic blend reshaped obesity-related gut dysbiosis and improved lipid metabolism in obese children
Source: Front Nutr. 2022 Aug 4;9:922993. doi: 10.3389/fnut.2022.922993 (PMC9386160; doi:10.3389/fnut.2022.922993)
Supplement: Supplementary file 1 [file Data_Sheet_1.pdf]

Supplementary Material

1 Supplementary Data

***In vitro* analysis for the multi-strain probiotic supplement in different forms**

To elucidate the capacity of reducing fat accumulation in intestinal cells, the human epithelial cell line Caco-2 (BCRC60182) was co-cultured with different forms of the multi-strain probiotic supplement. 0.4 µm pore insert wells were used to exclude direct contact of the Caco-2 cells with bacteria. Caco-2 cells were cultured at 37 °C under 5% CO_2 in Dulbecco's modified eagle medium (DMEM) supplemented with 10% fetal bovine serum (FBS). The cells were plated at a density of 2×10^5 cells per well into six-well plates and grown for 7 days in culture medium. The experimental medium was prepared as follows: the probiotic culture, powder, or product was added to 10 ml of DMEM containing 500 µmol/l oleic acid (OA) (Sigma-Aldrich, St. Louis, MO), and the pH was adjusted to 7.4. A final concentration of 2×10^8 cfu/ml probiotic strain in OA-DMEM was seeded on a Transwell membrane (SPL, Pochon, Korea) and inserted into a six-well culture plate containing Caco-2 cells. As the blank control group, solely DMEM was added to the Transwell without probiotic strain seeding. As the OA control group, DMEM containing OA was added to the Transwell without probiotic strain seeding. As the experimental group, Caco-2 cells were co-cultured indirectly with probiotic strains for 6 h, and collected under OA-treated conditions. Triacylglycerol (TG) was extracted from Caco-2 cells and TG quantification was performed according to the manufacturer's protocol (Cayman Chemical, Ann Arbor, MI).

The product form of multi-strain probiotic supplement retained the capability in reducing TG accumulation in Caco-2 cells.

OA is a common dietary unsaturated fatty acid in human diets, and leads to triglyceride (TG) accumulation in intestinal cells (Jang and others 2019). The direct contact of cells with bacteria was avoided by using Transwell system. The endogenous TG content was 76.5 ± 6.1% in no-OA treated Caco-2 control, and total TG content was 100 ± 2.7% in OA treated Caco-2 control. The supplementation of OA significantly induced TG accumulation in Caco-2 cells ($^{\#\#\#}P < 0.001$, **Fig S1**). The total TG contents were 82.8 ± 1.9 % ($^{***}P < 0.001$), 83.6 ± 1.0 % ($^{***}P < 0.001$), and 87.1 ± 0.9 % ($^{***}P < 0.001$) in AP-32, CP-9, and bv-77 treated Caco-2 cells, respectively. The mixture of these 3 probiotic strains was prepared in 3 different forms: microbial culture, spray-dried powder, and product with functional ingredient. These 3 forms of mixture significantly reduced the TG accumulation to 70.5 ± 6.2 %, 82.2 ± 0.1 %, and 74.3 ± 0.6 %, respectively in Caco-2 cells comparing to OA treated Caco-2 control ($^{***}P < 0.001$).

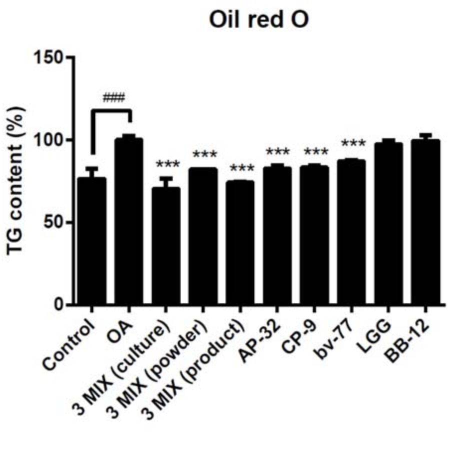

FIGURE S1 | Different forms of multi-strain probiotic mixture retained the capability in reducing oleic acid (OA)-induced intestinal triacylglycerol (TG) accumulation *in vitro*. OA increased TG accumulation in Caco-2 cells, and TG content was reduced by probiotic strains *L. salivarius* AP-32, *B. animalis* CP-9, and *L. rhamnosus* bv-77. The effect was retained in the mixture of these 3 strains in 3 different forms: liquid culture, spray dried powder, and the product containing functional ingredients (i.e., white kidney bean extract, psyllium husk, and Garcinia cambogia extract). Data are expressed as the mean ± SD from three independent experiments. Statistical comparisons were obtained by Student's t-test, $^{###}P < 0.001$ and $^{***}P < 0.001$. #: the comparison performed between control and OA groups. *: the comparison performed between OA and experimental groups.

Subjects were allocated randomly and baseline characteristics were compared between probiotic and placebo groups.

The baseline characteristics of every subject were recorded and no significant difference was found between placebo and probiotic groups except for systolic blood pressure (**Table S1**). The value of systolic blood pressure was 113.8 ± 3.1 mmHg and 124.7 ± 3.4 mmHg in probiotic and placebo groups, respectively ($^{*}P = 0.031$).

TABLE S1 | The baseline characteristics of subjects in probiotic and placebo groups.

**Parameter**	**Probiotics**	**Placebo**	***P*-value**
**Age** (years)	11.6 ± 0.6	11.2 ± 0.7	0.604
**Sex** (F/M)	13/14	10/16	0.583
**Acanthosis nigricans**	27	25	0.491
**Striae**	18	14	0.406
**Gynecomastia**	13	16	0.412
**Snore**	3	2	0.999
**Height** (cm)	149.8 ± 2.8	147.3 ± 3.1	0.533

**Weight** (kg)	68.2 ± 4.4	66.5 ± 4.6	0.824
**BMI** (kg/m^2)	29.7 ± 1.1	29.7 ± 1.0	0.790
**Waist circumference** (cm)	89.9 ± 2.7	90.4 ± 2.5	0.715
**Hip circumference** (cm)	98.0 ± 2.6	97.5 ± 2.6	0.873
**Systolic blood pressure** (mmHg)	113.8 ± 3.1	124.7 ± 3.4	*0.031
**Diastolic blood pressure** (mmHg)	72.9 ± 2.8	76.4 ± 2.7	0.277
**Heart rate** (beats/min)	88.9 ± 2.6	87.3 ± 2.7	0.986
**Biceps** (mm)	33.5 ± 1.4	32.1 ± 1.5	0.682
**Triceps** (mm)	44.1 ± 1.7	43.2 ± 1.9	0.650
**Subcapular** (mm)	46.4 ± 1.6	42.8 ± 1.6	0.135
**Thigh** (mm)	46.8 ± 1.6	45.3 ± 1.3	0.516
**T3** (ng/dl)	144.0 ± 4.7	151.1 ± 5.5	0.247
**T4** (ug/dl)	7.8 ± 0.5	8.4 ± 0.3	0.901
**TSH** (uIU/ml)	2.4 ± 0.3	3.0 ± 0.3	0.188
**Glucose AC** (mg/dl)	89.3 ± 1.2	89.7 ± 1.4	0.215
**HbA1c** (%)	5.6 ± 0.1	5.6 ± 0.0	0.548
**C-peptide** (ng/ml)	2.1 ± 0.2	2.4 ± 0.2	0.255
**Insulin** (uIU/ml)	13.8 ± 1.2	15.9 ± 2.2	0.887
**HOMA-IR**	3.1 ± 0.3	3.5 ± 0.4	0.838
**Total Cholesterol** (mg/dl)	172.3 ± 5.9	163.1 ± 7.3	0.157
**LDL** (mg/dl)	119.4 ± 5.0	104.3 ± 6.5	0.155
**HDL** (mg/dl)	45.6 ± 1.7	46.1 ± 2.1	0.510
**Triglyceride** (mg/dl)	96.5 ± 7.4	83.6 ± 7.9	0.144
**Uric acid** (mg/dl)	6.0 ± 0.2	6.1 ± 0.2	0.845
**GOT** (IU/L)	26.4 ± 2.7	27.1 ± 2.0	0.407
**GPT** (IU/L)	35.0 ± 6.8	34.5 ± 5.7	0.728
**Adiponectin** (mg/L)	7.1 ± 0.6	5.9 ± 0.6	0.154
**Leptin** (pg/ml)	2792.4 ± 175.1	2552.2 ± 131.9	0.367
**TNF-α** (pg/ml)	27.5 ± 9.2	19.4 ± 4.2	0.698

Data are presented as mean ± SEM of the results from every subject.

The Fisher's exact test was used to compare categorical variables.

The Mann-Whitney U test was used to compare continuous variables.

The intervention didn’t cause adverse effects and the probiotic supplement displayed hepatoprotective effects.

The number of people with acanthosis nigricans, striae, gynecomastia, and snore were not affected by the intervention in either placebo or probiotic group. Intriguingly, the supplement of probiotics significantly reduced serum liver enzymes, GOT and GPT.

TABLE S2 | The comparison of physiological and blood biochemical values obtained before and after the probiotic intervention.

**Parameter**	**Probiotics**	**Placebo**	***P*-value**
**Acanthosis nigricans** (N)			
Baseline	27	25	0.491
End	27	25	0.491
**Striae** (N)			
Baseline	18	14	0.406
End	19	13	0.166
**Gynecomastia** (N)			
Baseline	13	16	0.412
End	14	16	0.583
**Snore** (N)			
Baseline	3	2	0.999
End	2	3	0.669
**GOT** (IU/L)			
Baseline	26.4 ± 14.2	27.1 ± 10.1	0.407
End	20.9 ± 6.6	22.0 ± 6.8	0.728
Change rate (%)	86.3 ± 21.3**	84.1 ± 17.9***	0.575
**GPT** (IU/L)			
Baseline	35.0 ± 35.6	34.5 ± 29.3	0.728
End	23.5 ± 15.5	26.6 ± 18.2	0.689
Change rate (%)	85.8 ± 35.7*	87.2 ± 29.3**	0.755
**Uric acid** (mg/dl)			
Baseline	6.0 ± 0.2	6.1 ± 0.2	0.845
End	5.7 ± 0.2	5.8 ± 0.3	0.581
Change rate (%)	95.7 ± 1.9*	95.8 ± 2.6	0.999

Data are presented as mean ± SEM of the results from every subject.

Change rate: The percentage of end/baseline in every sample.
The Mann-Whitney U and Wilcoxon signed rank test were used to compare continuous variables.

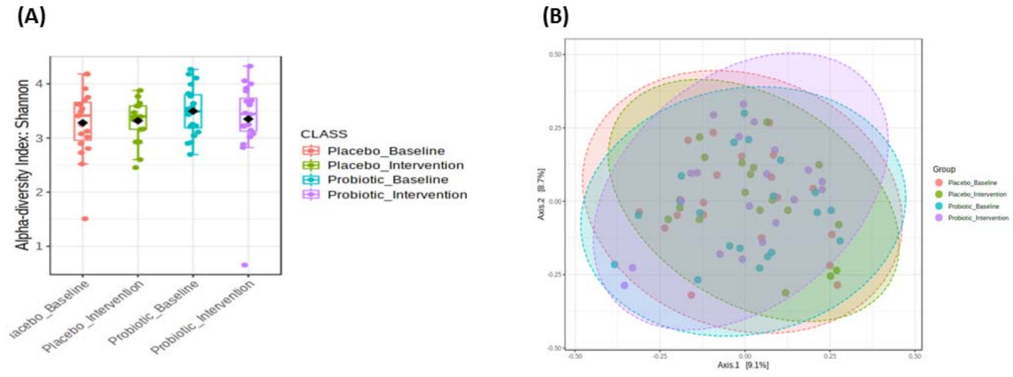

FIGURE S2 | No significant change was found in both alpha and beta diversity between the probiotic and placebo groups.

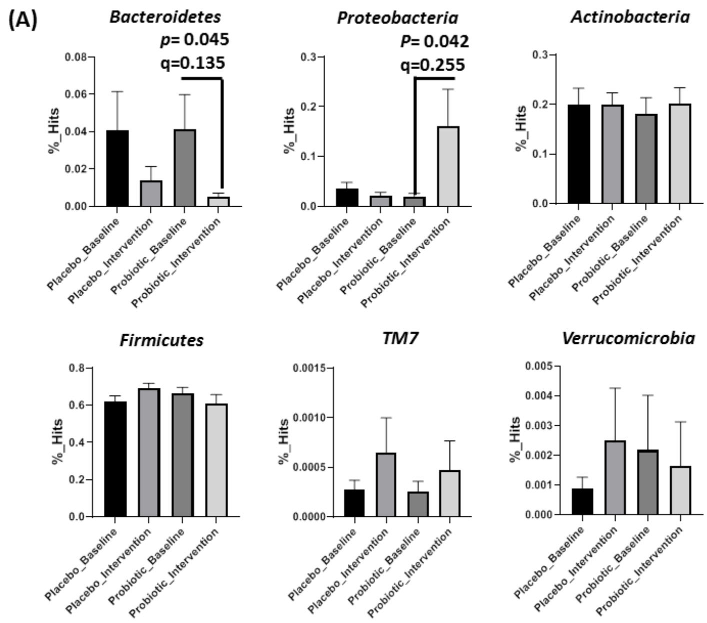

(B)

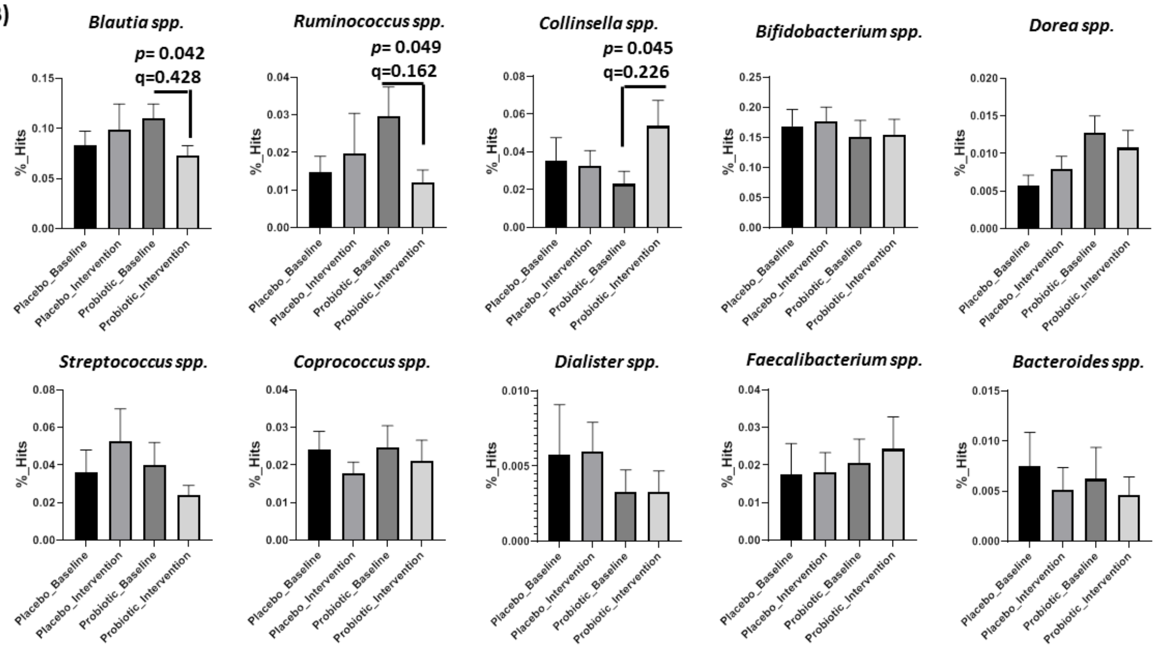

FIGURE S3 | (A) At phylum level, *Proteobacteria* (*p*=0.042, FDR adjusted *q*=0.255) increased and *Bacteroidetes spp.* (*p*=0.045, FDR adjusted *q*=0.135) decreased after the probiotic supplement. (B) At genus level, *Blautia* spp., *Ruminoccus* spp., *Streptococcus* spp. *Coprococcus* spp., *Dorea* spp. and *Bacteroides* spp. decreased in probiotic group, but the *Streptococcus* spp. *Coprococcus* spp., *Dorea* spp. and *Bacteroides* spp. didn't reach statistical significance. The FDR adjusted *p*-value is indicated as the *q*-value.

TABLE S3 | All the top 10 phylum and genus abundance and utilized unpaired t test with Welch's correction. The FDR adjusted *p*-value is indicated as the *q*-value

**Probiotic baselines vs. intervention**					**Placebo baselines vs. intervention**			
**Phylum**	**p-value**	**rank**	**q-value**		**Phylum**	**p-value**	**rank**	**q-value**
Proteobacteria	0.042	1	0.255		*Firmicutes*	0.075	1	0.450
Bacteroidetes	0.045	2	0.135		*Bacteroidetes*	0.233	2	0.699
Firmicutes	0.345	3	0.691		*Verrucomicrobia*	0.249	3	0.499
TM7	0.502	4	0.753		*TM7*	0.283	4	0.425
Actinobacteria	0.661	5	0.793		*Proteobacteria*	0.451	5	0.541
Verrucomicrobia	0.680	6	0.680		*Actinobacteria*	0.985	6	0.985
**Probiotic baselines vs. intervention**					**Placebo baselines vs. intervention**			
**Genus**	**p-value**	**rank**	**q value**		**Genus**	**p-value**	**rank**	**q-value**

Blautia	0.043	1	0.428		*Coprococcus*	0.277	1	>0.999
Collinsella	0.045	2	0.226		*Dialister*	0.406	2	>0.999
Ruminococcus	0.049	3	0.162		*Faecalibacterium*	0.507	3	>0.999
Bacteroides	0.215	4	0.537		*Bacteroides*	0.522	4	>0.999
Streptococcus	0.594	5	>0.999		*Blautia*	0.602	5	>0.999
Coprococcus	0.638	6	>0.999		*Ruminococcus*	0.671	6	>0.999
Faecalibacterium	0.686	7	0.980		*Dorea*	0.718	7	>0.999
Dorea	0.741	8	0.927		*Bifidobacterium*	0.820	8	>0.999
Bifidobacterium	0.916	9	>0.999		*Collinsella*	0.839	9	0.932
Dialister	0.931	10	0.931		*Streptococcus*	0.881	10	0.881

TABLE S4 | The relatively abundance of 11 genera and 5 species utilized unpaired t test with a Welch's correction. The FDR adjusted P value is denoted as the q value

**Placebo baselines vs. intervention**						
**Genus**	**p-value**	**Difference**	**SE of difference**	**t ratio**	**df**	**q-value**
Blautia	0.603	-0.183	0.347	0.526	27.51	0.954
Collinsella	0.889	0.061	0.435	0.141	26.5	0.954
Bifidobacterium	0.820	-0.049	0.215	0.230	35.07	0.954
Bacteroides	0.514	0.362	0.547	0.661	29.65	0.954
Lactobacillus	0.277	0.830	0.737	1.125	16.02	0.954
Streptococcus	0.944	0.036	0.506	0.070	30.53	0.954
Megamonas	0.648	-0.292	0.631	0.463	22.55	0.954
[Ruminococcus]	0.921	0.036	0.358	0.100	35.09	0.954
Ruminococcus	0.400	0.322	0.378	0.853	33.6	0.954
Lachnospira	0.226	-4.410	3.509	1.257	16.44	0.954
Prevotella	0.118	0.954	0.580	1.644	18.05	0.954
**Probiotic baselines vs. intervention**						
**Genus**	**p-value**	**Difference**	**SE of difference**	**t ratio**	**df**	**q-value**
Blautia	0.494	0.157	0.227	0.691	35.42	0.610
Collinsella	0.215	-0.594	0.470	1.264	32.47	0.610
Bifidobacterium	0.916	-0.026	0.246	0.106	37.78	0.925
Bacteroides	0.309	0.581	0.556	1.044	20.01	0.610
Lactobacillus	0.037	-1.482	0.670	2.212	22.76	0.240
Streptococcus	0.452	0.238	0.312	0.763	28.2	0.610
Megamonas	0.262	0.928	0.803	1.156	19.09	0.610

[Ruminococcus]	0.592	0.161	0.297	0.541	31.34	0.658
Ruminococcus	0.043	0.571	0.269	2.120	27.26	0.240
Lachnospira	0.388	0.819	0.926	0.884	17.79	0.610
Prevotella	0.380	0.869	0.968	0.898	19.48	0.610
**Placebo baselines vs. intervention**						
**Species**	**p-value**	**Difference**	**SE of difference**	**t ratio**	**df**	**q-value**
Blautia producta	0.443	0.26	0.336	0.779	25.54	0.834
Akkermansia muciniphila	0.858	0.11	0.600	0.180	31.99	0.867
Bifidobacterium animalis	0.518	-0.31	0.472	0.655	28.55	0.834
Bifidobacterium pseudolongum	0.660	-0.22	0.490	0.444	31.48	0.834
Bifidobacterium bifidum	0.245	0.45	0.376	1.189	26.51	0.834
**Probiotic baselines vs. intervention**						
**Species**	**p-value**	**Difference**	**SE of difference**	**t ratio**	**df**	**q-value**
Blautia producta	0.445	-0.377	0.486	0.776	26.72	0.512
Akkermansia muciniphila	0.115	0.609	0.365	1.669	15.42	0.194
Bifidobacterium animalis	0.002	-41.980	11.540	3.637	15.02	0.012
Bifidobacterium pseudolongum	0.014	-4.108	1.511	2.719	17.73	0.036
Bifidobacterium bifidum	0.507	0.446	0.662	0.673	26.21	0.512
